# Complex trait subtypes identification using transcriptome profiling reveals an interaction between two QTL affecting adiposity in chicken

**DOI:** 10.1186/1471-2164-12-567

**Published:** 2011-11-21

**Authors:** Yuna Blum, Guillaume Le Mignon, David Causeur, Olivier Filangi, Colette Désert, Olivier Demeure, Pascale Le Roy, Sandrine Lagarrigue

**Affiliations:** 1 INRA, UMR598, Génétique Animale, IFR140 GFAS, 35000 Rennes, France; 2 Agrocampus Ouest, UMR598, Génétique Animale, IFR140 GFAS, 35000 Rennes, France; 3 Agrocampus Ouest, Applied Mathematics Department, 35000 Rennes, France; 4 ITAVI, F-75008, Paris, France

## Abstract

**Background:**

Integrative genomics approaches that combine genotyping and transcriptome profiling in segregating populations have been developed to dissect complex traits. The most common approach is to identify genes whose eQTL colocalize with QTL of interest, providing new functional hypothesis about the causative mutation. Another approach includes defining subtypes for a complex trait using transcriptome profiles and then performing QTL mapping using some of these subtypes. This approach can refine some QTL and reveal new ones.

In this paper we introduce Factor Analysis for Multiple Testing (FAMT) to define subtypes more accurately and reveal interaction between QTL affecting the same trait. The data used concern hepatic transcriptome profiles for 45 half sib male chicken of a sire known to be heterozygous for a QTL affecting abdominal fatness (AF) on chromosome 5 distal region around 168 cM.

**Results:**

Using this methodology which accounts for hidden dependence structure among phenotypes, we identified 688 genes that are significantly correlated to the AF trait and we distinguished 5 subtypes for AF trait, which are not observed with gene lists obtained by classical approaches. After exclusion of one of the two lean bird subtypes, linkage analysis revealed a previously undetected QTL on chromosome 5 around 100 cM. Interestingly, the animals of this subtype presented the same q paternal haplotype at the 168 cM QTL. This result strongly suggests that the two QTL are in interaction. In other words, the "q configuration" at the 168 cM QTL could hide the QTL existence in the proximal region at 100 cM. We further show that the proximal QTL interacts with the previous one detected on the chromosome 5 distal region.

**Conclusion:**

Our results demonstrate that stratifying genetic population by molecular phenotypes followed by QTL analysis on various subtypes can lead to identification of novel and interacting QTL.

## Background

In the last decade, integrative genomics approaches that take into account genotypic, molecular profiling and complex traits in segregating populations have been developed to dissect the genetics of complex traits such as human diseases or economically important traits in livestock or plants. Combining QTL mapping and high throughput transcriptome data is proving useful for characterizing QTL regions and elucidating genes and biological pathways that affect complex traits [1-9]. The term "Genetical Genomics" or "Systems Genetics" refers to such a combinatorial approach.

One strategy commonly used by authors working in this context was based on the identification of genes having an eQTL that colocalizes with the QTL responsible for the complex trait of interest. Such a strategy considers the expression level of each gene available on a microarray as a quantitative trait and uses genetic markers to identify genomic regions that regulate gene expression phenotypes; these regions are named eQTL (expression Quantitative Trait Loci). The function of the gene that its mRNA level is controlled by a region can provide new functional information about the candidate gene sought in the eQTL region. Colocalization of eQTL with the QTL for complex trait can provide relevant information about the causative gene for the complex trait of interest. This strategy has been widely used in various species (flies [1,10], mice [2-4], rats [5], human [6], eucalyptus [7], Arabidopsis [8], livestock species [9,11] has been reported). When combined with mathematical modeling proposed by Schadt *et al*. [3], this strategy becomes very efficient for distinguishing causal from reactive genes for the complex trait and for identifying the "driver" genes and pathways that are responsible for a complex trait.

Another strategy is based on defining subtypes for a complex trait using gene expression profiles. It is well known that a population measured for a complex trait through one criteria (for example, Body mass index for obesity) may actually have distinct molecular subtypes for this complex phenotype. Use of gene expression profiles may allow the identification of such biologically distinct subtypes. The standard procedure is to identify genes whose expression is correlated to the complex trait and then perform a classification of individuals in order to observe specific subtypes. Applied on a segregating population, the identification of subtypes combined with QTL analysis performed for these subtypes can separately improve sensitivity of QTL detection and reveal new loci. This strategy was first performed by Schadt *et al*. (2003) [4] using a mouse population and then in 2009 by our team using a chicken segregating population [12]. In these two studies, two QTL were observed for the fat mass, one initially observed on the full F2 set and another one only observed when one subtype was removed. As illustrated by these studies, the core of the approach is the determination of subtypes within a segregating population on the basis of the genes correlated to the complex trait. In the present paper, we propose to identify these genes using a method called Factor Analysis for Multiple Testing (FAMT) which takes into account the hidden dependence structure that may result from population structure or/and technical artefact of gene expression profiling experiment, independent of the trait of interest ([11], [13]). We then show the utility of this method to define phenotype subtypes more accurately and to reveal interaction between 2 QTL.

## Results and discussion

### Identification of animal subtypes for fatness trait using the FAMT method

The first step was to identify which of the 11213 genes expressed in the liver were correlated to the trait of interest, the abdominal fat weight (AF), in the 45 related offspring's. Pearson correlation between hepatic transcript levels and AF trait identified 287 genes significantly associated at the nominal p-value of 0.05 without any correction for multiple tests. To increase the size of this list, Le Mignon *et al*. [12] added to this first list, genes significantly differentially expressed between the 10 leanest and fattest birds in the family. As such, a list of 660 genes was obtained with a significance threshold of 0.05 (Student's t-test p-value and Pearson correlation test p-value) without any correction for multiple tests. It should be noted that applying correction for multiple testing resulted in no gene being differentially expressed. This result might be explained by either a poor genetic variability between animals, which are half sib offsprings, or dependence between genes. Indeed, standard methods to find significant correlation between gene expressions and a variable of interest ignore the correlations among expression profiles [14]. This dependence structure leads to correlation among test statistics, which leads to under representation of the smallest p-values [15]. This can be explained by a number of unmeasured or unmodeled factors independent of the variable of interest (in our study, the AF trait) that may influence the expression of any particular gene ([16], [13]). These factors may induce additional variability in the expression levels and decrease the power to detect the true correlation with the variables of interest. Recently, several studies have introduced models taking into account this gene dependence. In particular, Friguet *et al*. [13] propose to model this sharing of information by a factor analysis structure in a method called Factor Analysis for Multiple Testing (FAMT). The estimated factors in the model capture components of the expression heterogeneity independent of the effects of the variable of interest. We applied this method to our data: 688 transcripts significantly correlated to the AF trait were identified taking into account the existence of six factors containing a common information shared by all genes and independent from the AF trait. The interpretation of these factors was analyzed and discussed in Blum *et al*. [11]. For the further analyses in the present paper, we subtracted the linear dependence kernel defined by the six factors from the 688 raw gene expressions to obtain 688 factor-adjusted expressions as in Blum *et al*. [11].

**Figure 1:**
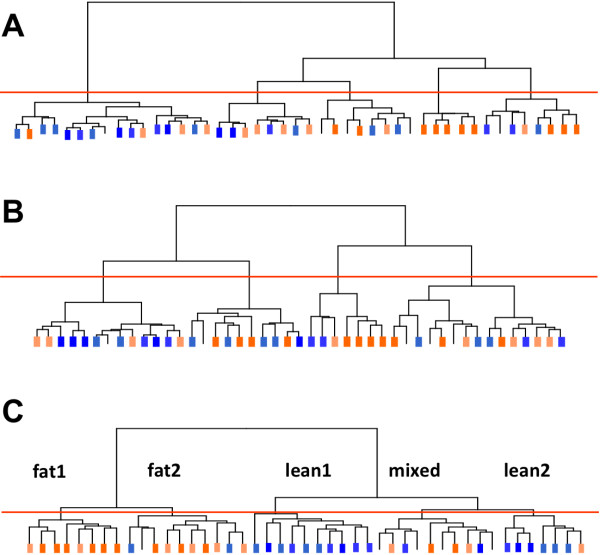
**Dendrograms of birds using different gene lists related to fatness and obtained by different statistical methods**. **(A) **287 genes correlated to the fatness trait obtained by a classical approach. **(B) **660 genes correlated to the fatness trait and/or differentially expressed obtained by classical approaches [12]. **(C) **688 genes correlated to the fatness trait obtained by FAMT method [11]. Orange/blue bars indicate the 20 fattest/leanest animals respectively. Darkest bars correspond to the 10 extreme animals of the 2 fattest/leanest groups. Colorless bars correspond to the 5 intermediate chickens.

The second step was to identify the best gene list that distinguishes potential subtypes for the AF trait within the 45 offspring. Separate hierarchical clustering of the birds was performed using either the 287 (Figure 1A) and the 660 genes obtained by classical methods in step one (Figure 1B), or the 688 genes obtained by the FAMT method (Figure 1C). For the latter we used the FAMT adjusted expression values in the clustering algorithm. The results of the three clusterings are shown in Figure 1. The set of 688 genes is clearly more efficient to separate fat and lean birds, and to generate different subtypes for the AF trait. As indicated in Figure 1C, this gene list allows us to clearly distinguish two subtypes for the fat birds and two other subtypes for the lean birds in addition to one subgroup mixing lean, intermediate and fat birds. This gene list includes almost all of the genes of the 287 genes (93%) but is twice as large. This larger number suggests that correlation between many gene expressions and the variable of interest is underestimated due to the hidden dependence structure. Finally, this gene list is quite different from the 660 gene set with only 69% common genes, suggesting a notable number of false positive genes in the latter due to the absence of correction for multiple testing and for gene dependence.

These results clearly show the importance of taking into account the gene dependence due to additional sources of variation, especially when the expression variation related to the variable of interest may be low and therefore easily impacted by these additional sources.

### A new QTL revealed by removing one of the two lean subtypes: genetic characterization of this subtype

**Figure 2:**
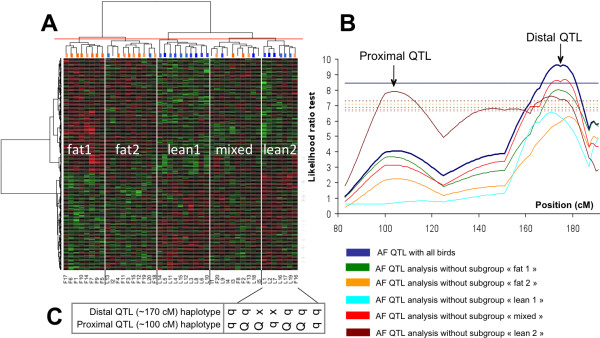
**HCA and linkage analysis using subtype combination**. **(A) **Heatmap using the 688 genes (Y axis) and the 45 chickens (X axis). **(B) **Interval mapping for the AF trait on chromosome 5, with the whole family (blue) and without one subgroup observed by HCA (other colors). The graph gives the statistical test (Likelihood Ratio Test) related to the test "no QTL" versus "one QTL" (y-axis") for every tested location on the chromosome (centiMorgan, x-axis). The chromosome-wide significance threshold at the 5% level obtained for the analysis including all animals (blue) and for analysis without one of the five subgroups: fat1 (green), fat2 (yellow), lean1 (light blue), mixed (red) or lean2 (brown) are displayed. The genetic distances (cM) and likelihood ratio test (LRT) are shown on the X-axis and Y-axis, respectively. **(C) **The two letters indicate the Q or q haplotype inherited from the sire, with a probability > 99% for the proximal QTL (first letter) or the distal QTL (second letter); × indicates a probability < 99%. The probability of inheriting the paternal Q versus q haplotype was calculated only on the basis of marker information in the region of interest.

Based on the clustering obtained using the FAMT adjusted expression (Figure 2A), we performed linkage analyses for the AF trait on the chromosome 5, either with the whole family or by removing successively one of the five subgroups (Figure 2B). As indicated in Figure 2B, the majority of analyses gave the same LRT curves with the expected AF QTL located around 168 cM on the chromosome 5 [12]. However, after removing the lean subtype called lean2, a new significant QTL (p-value < 0.05) was detected on a proximal chromosome 5 locus around 100 cM with an effect of 1.19 phenotypic standard deviation. Alternative and not necessarily exclusive hypotheses can be drawn to explain the detection of the second QTL after the exclusion of the lean2 group:

1) The first hypothesis is the presence of animals having an AF value in disagreement with the paternal Q/q haplotype in the excluded lean2 group. Removing such birds, especially when their AF values are extreme, can largely increase the power of QTL detection when the design analyzed has a low size. We determined for each offspring the Q/q haplotype corresponding to the proximal QTL (see Methods section). Two out of the 7 birds of the lean2 subtype have the Q paternal haplotype that contributes to a high fat mass (L2 and L7 birds) (Figure 2C). However, we can notice that the lean1 subtype is in the same configuration with 2 extreme lean birds as well (L4 and L5) with the Q haplotype at the new QTL (Table 1) but does not allow to reveal this latter after being removed.

**Table 1:** Haplotype determination for the distal and proximal AF QTL on chromosome 5.

Animal ID	Subgroup	Proximal AF QTL haplotype	Distal AF QTL haplotype	Both AF QTL haplotypes
L1	lean2	q	q	q-q
L2	lean2	Q	q	Q-q
L3	lean1	q	Q	q-Q
L4	lean1	Q	q	Q-q
L5	lean1	Q	q	Q-q
L6	lean1	q	x	X
L7	lean2	Q	x	X
L8	lean1	q	Q	q-Q
L9	mixed	q	q	q-q
L10	lean1	x	q	X
L11	lean1	x	q	X
L12	lean1	q	q	q-q
L13	fat2	x	Q	X
L14	lean1	Q	q	Q-q
L15	lean1	x	Q	X
L16	lean2	q	x	X
L17	lean2	Q	q	Q-q
L18	mixed	Q	Q	Q-Q
L19	lean2	Q	q	Q-q
L20	fat2	q	q	q-q
I1	mixed	x	Q	X
I2	fat2	x	Q	X
I3	mixed	q	q	q-q
I4	mixed	Q	Q	Q-Q
I5	mixed	Q	q	Q-q
F1	fat1	Q	Q	Q-Q
F2	mixed	Q	Q	Q-Q
F3	fat2	Q	Q	Q-Q
F4	fat2	Q	Q	Q-Q
F5	fat1	Q	x	X
F6	fat1	q	q	q-q
F7	fat1	Q	Q	Q-Q
F8	mixed	x	Q	X
F9	fat1	Q	x	X
F10	fat1	q	q	q-q
F11	fat2	x	q	X
F12	fat2	Q	Q	Q-Q
F13	mixed	q	q	q-q
F14	fat1	x	Q	X
F15	fat2	x	Q	X
F16	lean2	q	q	q-q
F17	fat1	x	Q	X
F18	fat2	Q	Q	Q-Q
F19	fat2	Q	Q	Q-Q
F20	mixed	q	Q	q-Q

		34 animals	40 animals	29 animals

Animal labels F1 to F20 indicate the 20 fattest chickens, L1 to L20 the 20 leanest chickens and I1 to I5 the 5 intermediate chickens.

The two letters indicate the Q or q haplotype inherited from the sire, with a probability > 99% for the QTL at 84 cM (first column) or the QTL at 168 cM (second column); x indicates a probability < 99%. The probability of inheriting the paternal Q versus q haplotype was calculated by QTLMap software only on the basis of marker information in the region of interest.

2) The second hypothesis is that the proximal and distal QTL on chromosome 5 interact with each other. In our specific case, this means that the allele configuration of the distal QTL at 168 cM influences the effect of the proximal QTL and therefore masks it when the whole family is used. To investigate this explanation further, we analyzed the paternal haplotype at the QTL around 168 cM for the different birds of the lean2 subtype (Figure 2C). We determined the paternal haplotype for 5 out of the 7 birds belonging to this subtype considering a probability > 99%. Interestingly, all of five birds have the same haplotype q. This observation suggests that the two QTL are interacting: the presence of the Q allele at the distal locus enhances the allelic effect at the proximal QTL and the presence of q allele at the distal locus weakens the allelic effect at the proximal QTL.

### Interaction testing between the proximal and distal AF QTL on chromosome 5

Considering a transmission probability greater than 99%, we determined the paternal haplotype for the proximal and distal QTL in 29 birds (40 and 34 birds for the distal QTL (at 168 cM +- 15 cM) and proximal QTL (at 100 cM +- 20 cM)) respectively as shown in Table 1.

**Figure 3:**
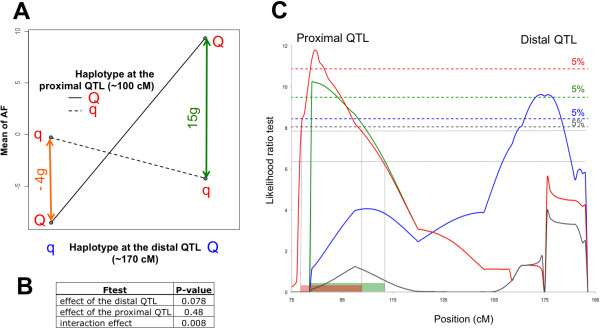
**Interaction testing**. **(A) **Interaction graph is given by a two-way analysis of variance considering the two QTL as two fixed factors with an interaction between them. The "Q-q" QTL effect at the proximal QTL is more important when the animals have the Q allele at the distal QTL (-4g/+15g with the q/Q allele at the distal QTL respectively). **(B) **The table gives the resulting p-value for both factors and the interaction. **(C) **QTL analysis using the "interaction model": the blue curve corresponds to the classical model testing the hypothesis « No QTL versus 1 QTL», the grey curve to the model testing «One QTL versus 2 QTL», the green curve corresponds to the interaction model « No QTL versus 1 QTL in interaction with another one fixed at 168 cM » and the red corresponds to the interaction model after adding the six novel markers. For each case, the significance threshold at the 0.05 level is displayed.

Using these 29 birds, we first performed a two-way analysis of variance considering the two QTL as two fixed factors with an interaction between them. As indicated in Figure 3A, the analysis shows clearly a significant interaction between the two QTL (p-value < 0.01). The difference between Q versus q for the proximal QTL is higher when the haplotype is Q at the distal QTL (+15g) than when the haplotype is q (-4g).

We also tested the QTL interaction using the QTLMap software with the "interaction model" ([17], [18]). The procedure tests the model: "No QTL" versus "1 QTL in interaction with another known QTL". We chose to set the location of the distal QTL at 168 cM, corresponding to the maximum LRT. Compared to the analysis of variance, the advantage of this QTL analysis is to set the location for only one of the two QTL presumed in interaction, increasing the number of birds analyzed (40 versus 29 animals) and then allowing to better localize the second QTL. As depicted in Figure 3B, the green curve corresponding to the interaction model analysis shows clearly a significant QTL in the proximal region (p-value < 0.05) in interaction with the fixed QTL at 168 cM. Furthermore, an additive model testing the hypothesis "one QTL" versus "2 QTL" does not highlight the proximal QTL (grey curve, Figure 3B), which is consistent with our expectation that the two QTL are in interaction.

To obtain a better estimate of the proximal QTL location, we developed six novel informative SNP markers in the proximal region at 67, 77, 80, 86, 89 and 95 cM respectively and genotyped the 40 animals accordingly. As indicated in Figure 3B, where the red curve corresponds to the interaction model performed with additional markers, the most probable position of the proximal QTL in interaction with the distal QTL on the chromosome 5 was found at 85 cM (p-value < 0.05) with a Confidence Interval (CI) from 78 to 102 cM.

Among the selected 688 genes, we identified 4 genes having a similar QTL profile as the abdominal fatness trait on the chromosome 5 (Table 2). These genes have a distal eQTL colocalizing with the AF distal region (observed by using the classical QTL additive model). They also have a proximal QTL colocalizing with the AF proximal region, with an interaction with the distal eQTL (p-value < 0.1 by using the "interaction model" of QTLMap software and the novel markers). Interestingly, one of these genes has the highest correlation with the AF trait (-0.58 Pearson correlation coefficient). Moreover, all 4 genes were differentially expressed (p-value < 0.1) between the two lean subtypes previously detected (lean1 and lean2). This observation can be interpreted as another illustration of the interaction effect between the proximal and distal AF QTL, but at the gene expression level. Taken together, these observations suggest that at least one of these 4 genes may be a signature of the causative mutation underlying the adiposity trait. These genes produce unknown proteins and/or proteins not particularly related to the adiposity. Further investigations will be necessary to confirm such a signature and clarify the role of these genes in lipid metabolism and adiposity.

**Table 2:** Genes for which RNA level is controlled by the two proximal and distal regions in interaction similarly to the adiposity phenotype

			Classical Model		Interaction Model		Test Lean1/lean2	
**oligo ID**	**HGNC**	**corr**	**location**	**maxLRT**	**location**	**maxLRT**	**DE**	**p-value**

RIGG00027	BPNT1	-0.36	182	9.6*	95	9.8^+^	-/+	+
RIGG05332	NULL	-0.39	178	6.3^+^	77	11.5*	-/+	*
RIGG07405	P4HA2	-0.38	185	7.3^+^	73	12.0*	-/+	+
RIGG12578	NULL	-0.58	158	11.7*	86	9.3^+^	+/-	+

For each gene, are given the oligonucleotide identifier (oligo ID), the HGNC abbreviation (HGNC), the Pearson correlation with the abdominal fatness (corr), the maximum LRT location in cM and the maximum LRT (maxLRT) using either the classical model or the interaction model. The two last columns are related to the statistical test comparing the expression between the lean1 and the lean2 subtypes: the differential expression between the two subtypes (DE = lean1/lean2) and the p-value associated are indicated. * and +: p-value at the chromosome level <0.05 and <0.1 respectively.

## Conclusion

In this study, we show the value of determining phenotype subtypes underlying a complex trait by using gene expressions. This subtype identification combined with QTL mapping improves the characterization of QTL responsible for adiposity, by revealing a new QTL in interaction with a previous one. This study also highlights the interest to use FAMT procedure to define more accurately these subtypes for a complex trait compared to classical methods. At the core of the approach proposed here is the phenotype subtype identification, which is still rarely used in the Genetical Genomics field and was reported once a few years ago by Schadt and colleagues [4]. In our report we show the advantage of using such approach in revealing interaction among QTL and discovery of new QTL underlying complex traits.

## Methods

### Animal design and microarray setup

Animal design, genotyping and microarray setup are previously described by Le Mignon *et al*. [12]. Briefly, the animal design corresponds to 45 male offspring produced by a sire known to be heterozygous for a QTL affecting abdominal fatness (AF) on chromosome 5 with a location confidence interval extended from 156 cM to 187 cM and a significant effect of 1.03 phenotypic standard deviation. This sire are not heterozygous for other AF QTL on GGA1, GGA3 and GGA7 previously detected in a three-generation F0-F1-F2 design performed by intercrossing two experimental chicken lines divergently selected for abdominal fatness from which the sire has been produced. Genotyping for GGA5 chromosome was performed for 10 markers (ADL0292, ADL0023, MCW0238, ADL0233, MCW0026, SEQF0079, SEQF0080, SEQF0082, SEQF0085, ROS330 at 83, 100, 125, 151, 162, 166, 175, 187, 190, 192 cM respectively). Markers were chosen from available markers [19] or developed for this program [12]. The six additional SNP markers were developed from the chicken genome sequence assembly and correspond to rs15678496, rs15683152, rs15685956, rs16689818, rs15691594, rs14531246 at 67, 77, 80, 86, 89 and 95 cM respectively. Gene expression measurements were obtained from the livers of these animals using a 20 K chicken oligo array (Ark-genomics). 11213 genes (55 % of the 20461 genes) were selected as expressed in the liver. The raw and normalized microarray data were deposited in the Gene Expression Omnibus (GEO) public repository [20]. The accession number for the series is GSE12319 and the sample series can be retrieved with accession numbers GSM309564 to GSM309609.

The animal labels were defined as follows: F1 to F20 for the 20 fattest animals, L1 to L20 for the 20 leanest animals and I for the 5 intermediates.

All experiments were conducted under Licence N°; 37-123 from the Veterinary Services, Indre et Loire, France and in accordance with guidelines for care and use of animals in Agricultural Research and Teaching (French Agricultural Agency and Scientific Research Agency).

### Classical expression analysis

As the variable of interest in the biological study is continuous, we calculated the Pearson correlation coefficient for each gene expression and deduced the number of genes correlated to the trait by considering the p-values under the cutoff 0.05. To control the False Discovery Proportion (FDR) we performed the Benjamini-Hochberg correction for multiple testing [21].

### Factor analysis method

The method takes into account the gene dependence structure and consequently, the impact of dependence on the multiple testing procedures for high-throughput data. Indeed, genes can have similar expression profiles because they are involved in common pathways but independently of the variable of interest (AF in our case). The common information shared by all the variables (i.e. gene expressions) and independent of the variable of interest is modeled by a factor analysis structure. An EM algorithm is used to estimate the model. Once the factor model is estimated, factor-adjusted test statistics are obtained by correction of the classical tests from the effect of the common factors. David Causeur's team showed that the resulting tests statistics are asymptotically uncorrelated, which improves the overall power of the multiple testing procedure ([13], [22]). The algorithm is implemented in the "FAMT" R package available from CRAN. As in Blum *et al*. [11], the raw expression data set is adjusted for the estimated independent factors, which results in the so-called factor-adjusted expression data.

### QTL and eQTL mapping

QTL (eQTL) mapping consists in mapping on the genome, regions that control the variation of a complex trait (expression trait). Before QTL analyses, the AF trait values of the sire family (71 birds) were adjusted for hatch and dam effects by two-way variance analysis, including body weight at slaughter as a covariate (SAS GLM procedure). For the eQTL analyses, no adjustment of the gene variables was performed for hatch and dam effects because of the small size of the population studied (45 birds). QTLMap software based on an interval mapping method described by Elsen *et al*. [23], was used to detect QTL (or eQTL) affecting the AF trait (or a gene expression phenotype). The statistical variable for testing the presence of no QTL (or no eQTL) versus one QTL (or one eQTL) at one location and also of one QTL versus two, was an approximate likelihood ratio test (LRT) [24]. Significance thresholds were empirically determined for AF QTL and transcript level eQTL from 2000 simulations performances assuming a polygenic model with a given heritability (h^2 ^= 0.5). The widely used "one LOD drop-off method" was applied to obtain 95% confidence intervals of the QTL location [25]. QTLMap software was also used to test an interaction between the proximal and distal QTL using the "interaction model" testing the hypothesis « No QTL versus 1 QTL in interaction with another one fixed in our study at 168 cM » [18]

We considered that a gene has an eQTL colocalizing with an AF QTL if the CI of the eQTL region was overlapping the CI of the QTL region.

## List of abbreviations

AF: Abdominal Fatness; FAMT: Factor Analysis for Multiple Testing; GGA: Gallus Gallus; eQTL: Expression Quantitative Trait Locus; CI: Confidence Interval; GEO: Gene Expression Omnibus; HCA: Hierarchical Cluster Analysis; HGNC: HUGO Gene Nomenclature Committee; EM: Expectation Maximization; LRT: Likelihood Ratio Test.

## Authors' contributions

GLM, CD and SL provided the transcriptomic data set. FP and OD performed genotyping. YB and GLM analyzed the expression data sets supervised by SL and DC. YB and GLM carried out the QTL and the eQTL mapping analyses supervised by SL, PL, OF and OD. SL and YB drafted the manuscript. SL supervised the project. All authors read and approved the final manuscript.
